# Sex and Gender Multidimensionality in Epidemiologic Research

**DOI:** 10.1093/aje/kwac173

**Published:** 2022-10-04

**Authors:** Greta R Bauer

**Affiliations:** Epidemiology and Biostatistics, Schulich School of Medicine & Dentistry, Western University

**Keywords:** bias, gender identity, gender role, methods, misclassification, sex characteristics, validity

## Abstract

Along with age and race, sex has historically been a core stratification and control variable in epidemiological research. While in recent decades research guidelines and institutionalized requirements have incorporated an approach differentiating biological sex from social gender, neither sex nor gender is itself a unidimensional construct. The conflation of dimensions within and between sex and gender presents a validity issue wherein proxy measures are used for dimensions of interest, often without explicit acknowledgement or evaluation. Individual-level dimensions of sex and gender are outlined as a guide for epidemiologists. Two case studies are presented. The first demonstrates how unacknowledged use of a sex/gender proxy for a sexed dimension of interest (uterine status) resulted in decades of cancer research misestimating risks, racial disparities, and age trends. The second illustrates how a multidimensional sex and gender framework may be applied to strengthen research on COVID-19 incidence, diagnosis, morbidity and mortality. Considerations are outlined, including 1) addressing the match between measures and theory, and explicitly acknowledging and evaluating proxy use; 2) improving measurement across dimensions and social ecological levels; 3) incorporating multidimensionality into research objectives; 4) interpreting sex, gender, and their effects as biopsychosocial.

Along with age and race, sex has historically been a core stratification and control variable in epidemiological research. For example, among 34 published Original Contributions in the *American Journal of Epidemiology* in the first half of 2020, 33 (97.1%) included sex or gender in some way. Sex/gender was used extensively in study design, including sample restriction (47.1%) or matching (5.9%), and in analysis. In unrestricted samples, sex/gender variables were used to describe the sample (77.8%), as covariates for adjustment (66.7%), and in effect-measure modification or stratified analyses (55.6%). Despite widespread use, sex/gender variables were rarely defined, though information was sometimes provided (e.g., "cisgender or transgender men", "pregnant women") or inferable.

Distinguishing between biological sex and social gender has been identified as critical to validity within epidemiological research (1), to reporting of results (2–4), and for science broadly (5). Recent changes to research policy and practice have institutionalized sex and gender. National Institutes of Health funding requires incorporation of sex as a biological variable (SABV) in clinical research (6–8), the Canadian Institutes of Health Research requires sex- and gender-based analysis (SGBA) (9,10), and the World Health Organization requires a gender

ORIGINAL UNEDITED MANUSCRIPT

perspective (11). “Two-step” measures have been increasingly incorporated into survey research, including one item each for sex and gender, allowing for sex versus gender distinction and the identification of gender minorities (12–14).

Despite these advances, conflation of sex and gender remains common in primary data collection, and administrative and survey data sources may add confusion by containing undifferentiated sex/gender measures. Undifferentiated data may be obtained based on self-report, administrative classification (e.g., insurance records, drivers’ licenses), or interviewer assessment of appearance or voice. While such classifications create interpretation challenges in disaggregating sex from gender, even two-step measures do not provide the information needed for a rigorous approach to causal questions. They commonly include a single dimension of sex (sex assigned at birth) and of gender (gender identity) (13). Common conceptualizations of sex and gender are multidimensional, with sex including anatomy and physiology, and gender including identity, expression, roles, and institutional gender, as well as experiences of gender-based stigma and discrimination (1–3,15). Health can be impacted by multiple dimensions of sex, multiple dimensions of gender, and their interactions (1,16–18). Thus, the question is not just sex versus gender, but one dimension of sex versus another, and one dimension of gender versus another. Secondary data analysis then often requires using a sex or gender measure that serves as a proxy for dimensions of interest, while primary data collection provides opportunities to identify and measure relevant dimensions.

## THE MULTIDIMENSIONALITY OF SEX AND GENDER

Multidimensionality in key sociodemographic measures is not new. Scholars of racial and ethnic disparities have long recommended disaggregating ethnic background, identity,

ORIGINAL UNEDITED MANUSCRIPT

community membership, administrative status, street race (the race others perceive one to be), experiences of interpersonal racism, and structural racism or colonialism (19–24), though practice lags behind recommendations (25). Sexual orientation is understood to encompass identity, behavior, and attraction (26), which differ from experiences of homophobia or biphobia, internalized homo- or bi-negativity, and structural heterosexism. Socioeconomic status incorporates education, occupation, wealth, resource access, and income across the life course (27). While the multidimensionality of sex and gender is well-theorized in feminist sociology, psychology, and sexology (16,17,28–32), in practice many epidemiologists are still learning to make clear distinctions between biological sex and social gender. Different dimensions are conflated or intertwined within most research, even when researchers focus specifically on sex and gender (16). In epidemiology, greater disaggregation of dimensions of sex and gender has the potential to increase measurement validity and improve causal understandings.

Table 1 presents a conceptual tool for researchers displaying some individual-level dimensions of sex, gender, undifferentiated sex/gender, and gender minority classification that may affect health. With the exceptions of sex assigned at birth (an event recorded on the original birth certificate) and chromosomal sex, all other dimensions may change over the life course. Sex as an overall domain also includes sexed anatomy (e.g., testes, breasts, placenta), physiology (e.g., endogenous and exogenous hormones), and intersex conditions. Sexed characteristics may be impacted by social context, but can generally be directly measured with validity across contexts.

Gender includes identity (gender identity, intersex identity), as well as lived or expressed gender, gender roles, and masculinity or femininity. It also includes metaperceived gender, the gender one knows others see them as, enacted gender discrimination and internalized stigma, and

ORIGINAL UNEDITED MANUSCRIPT

personally held gender ideology. As a social construct, understandings are specific to time, place, and culture, and may encompass ceremonial roles, longstanding cultural genders or time period-specific identities. Thus, gender measures may encode cohort-limited or culture-specific understandings that may not apply across contexts.

Sex/gender is included as a domain representing the conflation or lack of clear disaggregation of sex and gender present in undifferentiated survey data or in institutional gender (e.g., insurance databases). Increasing use of artificial intelligence to gather data (e.g., gender classification from image recognition software) provides another example of an undifferentiated measure with noted classification validity issues, particularly for dark-skinned women (33,34).

Gender minority status is included as a domain representing gender identity or expression that differs from what would be typical based on sex initially assigned at birth. Two-step cross-classifications are often used to define the population of those who are transgender, non-binary or gender-diverse (12,13,35). Additional aspects of minority gender may be identified under dimensions of gender identity, or on metaperception as gender minorities. For trans and other gender minority people, health and safety are heavily influenced by transphobic social responses to contravention of gender norms (36–38). While in research sexual orientation is typically considered a sexual minority status separate from gender minority status, this distinction is a cultural one not universally shared, for example within Indigenous cultures (39,40). Moreover, the health of sexual minorities globally is shaped by gendered expectations; for example in being targeted for violence based on breaking of gender norms or perceptions of gender nonconformity (41).

The final row in Table 1 contains sex- or gender-associated factors that may be considered sexed or gendered characteristics, or that differ on average across sex or gender groups; these

ORIGINAL UNEDITED MANUSCRIPT

include biological, psychological, behavioral, interpersonal, and social factors. The roles of such factors as direct or mediating causes may need to be clarified before developing sex- or gender-based strategies. Doing so can help mitigate risks of sex/gender essentialism. As an example, debate over SABV in clinical research has focused in part on the risks inherent in overdetermination of sex and development of "pink versus blue medicine" approaches. This has played out in practice, with the Food and Drug Administration instituting sex-based dosing for the sleep drug zolpidem (42), following documentation of excess adverse events in women tied to sex differences in metabolism. Ultimately, these differences were mediated primarily by body weight (43). In such cases, sex-specific dosing risks resulting in too high a dose for small men or too small a dose for large women.

Considering the broad multidimensionality of constructs often consolidated under "sex" or "gender" provides an opportunity to more precisely identify target populations, theorize causal pathways, and measure pertinent dimensions. Moreover, it makes explicit the use of proxy variables as substitutes for dimensions of primary interest. Approaching measurement validity epidemiologically opens up the possibility of evaluating performance of both direct measures and proxies.

ORIGINAL UNEDITED MANUSCRIPT

## CISNORMATIVITY AND RESEARCH

Cisnormativity is the assumption that all dimensions of sex and gender are concordant within individuals and consistent over the life course (44). While often used to describe assumptions applied to transgender persons, cisgender (non-trans) people commonly encounter cisnormative assumptions, such as that young women will have a uterus and be able to become pregnant. Under cisnormative assumptions, knowing one dimension about an individual, for example their lived gender presentation, allows for extrapolation to other dimensions such as their anatomy and gender role. For researchers, cisnormativity represents a fundamental failure of imagination. Overlooking opportunities to theorize and capture the multidimensionality of sex and gender in research measures and interpretations forecloses the possibility of a sexed and gendered understanding of both health disparities and causation. Below are presented two case studies, one historical example wherein use of an implicit proxy measure for the sex dimension of interest led to biased findings in reproductive cancer research, and one more recent COVID-19 example briefly illustrating the potential of multidimensional conceptualization of sex/gender differences in incidence, diagnosis, and severity or mortality to improve the specification of research hypotheses and measures, with the end goal of producing more valid findings of greater relevance to causation and intervention. .

**CASE STUDY 1: SEX/GENDER AS A PROXY FOR UTERINE STATUS**

Over 40 years ago in the *American Journal of Epidemiology*, the argument was made that studying cervical, endometrial and uterine corpus cancers among women underestimated risk, and the population needed to be corrected to reflect only those with uteri (45–47). Some researchers immediately began noting this as a limitation of their research, and suggesting the potential for bias in measures of association as well as incidence (48). However, existing data did not allow for use of uterine status to define sex strata, nor for corrections to the category of "women" using data from other sources. We can consider "women" versus "men" here an undifferentiated measure of sex/gender, variably defined depending on data source (e.g., surveys, medical records). Even in the context of increasing hysterectomies, there was not good data on the dimension of sex of primary interest: uterine status.

ORIGINAL UNEDITED MANUSCRIPT

Data would eventually show that hysterectomies had not only increased (and later decreased) over time, but varied by race/ethnicity, age, and geographic area. Differences in hysterectomy status that had been observed across single sociodemographic categories sometimes interacted. For example, while in much of the U.S., Black women were more likely than white women to have had a hysterectomy, in parts of the South white women were more likely (49).

While a few researchers in the 1990s or 2000s made corrections to analyses they or others had done (50–52), only more recently did the issue gain prominence (53), leading to a surge in hysterectomy-corrected analyses for Pap smears and cervical, endometrial, and uterine cancers (49,54–59). In total, hysterectomy corrections have decreased estimates of unmet Pap smear need (51) and increased incidence of cervical (56,58), endometrial (50,55), and uterine corpus (49,59) cancers, as well as cervical cancer mortality (54). Changes to risk estimates were substantial; for example, uterine cancer incidence estimates increased 30 to 100% across U.S. states (49). Associations were also altered, including age trends for cervical cancer (56,58), and cervical cancer screening (51), and racial/ethnic comparisons (49,50,54,55,57). The higher hysterectomy prevalence among Black versus white women in the U.S., for example, resulted in larger correction factors for Black women. This shifted understanding of racial disparities, shrinking differences in white-predominant outcomes such as endometrial or uterine cancer incidence (50,57). and increasing differences in Black-predominant outcomes such as cervical cancer incidence (57,58). Again, changes were substantial; the disparity in cervical cancer mortality was found to have been underestimated by 44% (54).

While the focus has been on *post hoc* correction of the denominator in incidence measures, the observed need to vary correction factors across different sociodemographic and time

ORIGINAL UNEDITED MANUSCRIPT

distributions highlights this as an issue of measurement validity. *Post hoc* corrections would not be needed if the population were stratified initially on the relevant measure of sex: uterine status. Moreover, *post hoc* corrections will not work well with analyses more complex than cross-tabulations, such as multifactorial causal analyses.

What if sex/gender were to be explicitly evaluated as a proxy for uterine status? While hysterectomy would be the biggest contributor to discordance between these two measures, additional discordance would result from some intersex conditions (women born without uteri), transgender women without uteri, and transgender men who retained their uteri. Using data from the National Health and Nutrition Examination Survey (NHANES) (60) to produce original estimates of intersection-specific hysterectomy prevalence (methods in Web Appendix 1; estimates in Web Table 1), in combination with published findings from the U.S. Census (61) and studies of transgender people (62,63), we can roughly approximate what such test performance might look like. Figure 1 displays estimates for sensitivity (panel 1A) and specificity (1B) for database-encoded sex/gender as a proxy for uterine status. Estimation calculations are described in Web Appendix 2, including formulae (Web Table 2), resulting numeric estimates (Web Table 3), and a summary of data sources used (Web Table 4).

Here sensitivity represents the proportion of those with uteri who are database-encoded women, and specificity the proportion of those without uteri who are database-encoded men. We see that sensitivity is good, but not perfect. In contrast, specificity is moderate and decreases sharply with age, more so among Black persons than white persons. Thus, positive predictive value of database-encoded sex/gender for predicting uterine status will also decrease sharply with age.

ORIGINAL UNEDITED MANUSCRIPT

This case provides a concrete example where cisnormative assumptions allowed the ongoing production of biased results by assuming that a sex/gender measure provided, or served as a valid proxy for, the dimension of interest: uterine status. The estimation of sensitivity and specificity shows how proxy validity for a biological sexed dimension was context-dependent, and varied across other variables and intersections of interest, introducing bias into associations as well as incidence estimates. The net impact was misestimation of cancer incidence, age patterns, and Black-white racial disparities in the U.S. While this example involved an outcome directly dependent on the sexed dimension of interest, bias would similarly be introduced in other sex/gender restricted or stratified analyses where use of a proxy altered the risk for the outcome.

## CASE STUDY 2: SEX/GENDER DIFFERENCES IN COVID-19 MORBIDITY AND MORTALITY

In the early months of the COVID-19 pandemic, observations were made of approximately comparable confirmed case numbers for women and men, but an excess in both intensive treatment and case fatality among men (64–67). These observations generated calls for deeper engagement with sex and gender in data collection, analysis, and reporting (68–70), and sex/gender-specific data trackers were coded to track data internationally (71,72). The excess risk of severe disease and mortality among men was confirmed in subsequent data tracking (71) and meta-analysis (73).

Observations of sex or gender differences are informative, but with insufficient theorization, cisnormativity often results in a default collapsing of multiple dimensions within and across sex and gender into undifferentiated or unclear measures, limiting the potential to

ORIGINAL UNEDITED MANUSCRIPT

explain health phenomena or provide specific guidance on interventions. To illustrate how it could guide measurement in an emerging area of inquiry, Table 2 applies the tool in Table 1 to consideration of sexed and gendered dimensions that were hypothesized to affect SARS-CoV-2 infection, diagnosis, and prognosis (severe disease or death). Gendered factors known to affect health care seeking or access were included, as they affect the probability of diagnosis. Given this case study's basis in early hypotheses published in 2020, we note that there are dimensions for which information was and is scant; for example, little is known about immune responses to infection among those who are transgender or intersex (74). We note that no hypotheses in Table 2 were based on undifferentiated sex/gender, highlighting how undifferentiated measures serve as proxies for more specific dimensions.

This example illustrates the usefulness of multidimensional conceptualization early in the research process, to identify relevant dimensions (avoiding use of proxies) and to consider causal pathways between them. For example, angiotensin-converting enzyme 2 (ACE2), the cell surface receptor used for SARS-CoV-2 cellular entry, is encoded genetically on the X chromosome, is downregulated by estrogen, and is expressed differentially in different tissues (including sexed organs) (75). Over the life course, gendered interactions and social norms also affect sexed bodies; a man's decision to not act to prevent SARS-CoV-2 transmission may be based in cultural norms of masculinity, as might his response to physical symptoms if infected. While commentaries reviewed for this case study focused on individual-level sex and gender measures, we know that sex and gender can shape epidemics through effects on susceptibility, exposure, diagnosis, treatment, and sequelae over the life course (76), and these may interface with sociostructural factors. For example, appropriate fit of personal protective equipment for health

ORIGINAL UNEDITED MANUSCRIPT

workers is shaped by the prominence of standard design based on measurements from young predominantly white and male U.S. military recruits in the mid-20th century (77).

While morbidity and mortality were the focus of this case study, sex and gender dimensions may also play a role in treatment outcomes (78), mental health (79), vaccine efficacy and uptake, COVID-19 complications and recovery, and long-term health impacts of both COVID-19 and the pandemic response (67,68,70,80). Direct measurement of relevant sex and gender dimensions and associated factors could allow for more nuanced research on SARS-CoV-2 infection and the impacts of the pandemic, with greater potential for improving results including long-term individual and social outcomes.

## CONSIDERATIONS FOR INCORPORATING SEX AND GENDER MULTIDIMENSIONALITY INTO RESEARCH

Addressing the issues of multidimensionality and measurement validity described here requires a shift in perspective that promotes deeper inquiry into both concepts and measurement. Advancing sex and gender in epidemiological research will involve addressing the match between measures and theory, improving measurement across dimensions and social ecological levels (e.g., individual, interpersonal, community, societal levels), incorporating multidimensionality in research objectives, and interpreting results in a biopsychosocial context that acknowledges that biology shapes and is shaped by social context and behavior.

### Addressing the match or mismatch between existing measures and theory

While differences between domains of sex and gender, and indeed many of the dimensions within each, have been acknowledged, implicit assumptions that one measure serves as a

ORIGINAL UNEDITED MANUSCRIPT

reasonable proxy for another are common. Even with precise specification, secondary data analysis limits available measures, and primary data collection of some biological measures may be expensive or invasive, potentially limiting sample size or creating selection bias. In these cases, explicitly differentiating between the dimension(s) of interest and the one(s) measured makes the use of a proxy variable explicit. Researchers can then report what is known about the relationship between the dimensions, and whether that varies across factors such as ethnicity, age, or exposure. This basic information may inform interpretation of results, make limitations clear, and better guide future research.

Established methods for evaluating the validity of measures have rarely been applied to sex and gender measures or their proxies. Just as gender is shaped intersectionally by race, ethnicity, and social class (81,82), sex and gender measurement validity across these groups must be evaluated to avoid inducing or distorting associations due to differential validity. Given their widespread use, it is especially important to understand how common measures in multi-user population data sets perform as proxies for other dimensions of causal interest. It will also be important to consider whether misclassification is differential or non-differential, not only for sex or gender exposure variables, but because of their common use in adjustment for confounding. With confounding, differential misclassification may produce bias in any direction, while non-differential misclassification will result in partial control of confounding, provided there is no qualitative interaction between exposure and confounder (83).

**Improving measurement across dimensions and social ecological levels**

Limitations in validity may be more profound for gender-related measures and group-level measures than for individual sex-related measures. Beyond self-reported gender identity, existing

ORIGINAL UNEDITED MANUSCRIPT

gender measures traverse adherence to gender norms, gender ideology, gender role conflict, and experiences of sexism (84). Because gender is cultural, measures attempting to capture masculinity and femininity vary in content and may not hold up well over time (84,85). Moreover, a dominant focus on either biological or psychosocial variables within existing data sets likely limits opportunities for secondary analyses across sex and gender dimensions. While researchers have attempted to remedy gender data deficiencies, for example creating a gender index from available childcare, employment, and education data (86), there is a clear need to not only improve measures, but to incorporate them in multi-user data sets.

While this paper highlights individual-level measurement issues, gender is multilevel (15,17), traversing social ecological levels from the individual to society. An integrated sex and gender approach will also need to also consider group-level factors, which may have consequences over the life course (15). These may include aggregate variables, for example community-level frequencies of beliefs regarding gender roles, or group-level factors such as policies that do not have individual-level analogues. Policies may be sex-related (e.g., state-level abortion policies) or gender-related (e.g., school-level gender identity inclusion policies). Researchers may find social ecological models (87,88) or an ecosocial perspective (89) helpful in integrating across levels, and including structural sexism, heteronormativity, or cisnormativity. Multilevel analysis of structural sexism and physical health has shown, for example, that more sexism at the U.S. state level was associated with worse health among women and men, but at the marital dyad level with worse health among women and better health among men; at the individual level (internalized sexism) there was no association (90).

**Incorporating multidimensionality into research objectives**

ORIGINAL UNEDITED MANUSCRIPT

The historic field of “sex differences research” (91) and “inclusion-and-difference” approaches to inclusiveness in science (92) have produced an overemphasis on describing and testing differences across sex/gender categories, at the expense of concerns over socially mediated gender inequalities, or even sex or gender equivalence (15,85,91,93). That meta-analyses in psychology have repeatedly documented gender differences to vary by age or be limited to particular ethnoracial groups (94–96) highlights the need for intersectional approaches that do not assume population homogeneity, or that health at different intersections of social identities/positions can be understood by combining the mean effects of each identity/position (97–100). It also highlights the importance of analyzing within-group heterogeneity to avoid “the tyranny of the mean”, an overemphasis on measures of central tendency and their differences (101).

Where sex or gender differences or effect-measure modification by sex or gender are observed, explanatory processes can involve multiple dimensions of sex, gender, or associated factors. Anticipation of which factors might explain such findings can allow for planned measurement of those variables. Explanatory analyses are important to strengthen causal understanding, generate theory, and counter sex/gender essentialism. Analyses of postulated mediating processes can provide supporting evidence for causal sex or gender effects and inform intervention strategies (102,103). For causal research, the presence of sex or gender in a directed acyclic graph (DAG) should lead to questions of which dimension(s) are truly playing causal roles, which may result in a better-specified DAG and ultimately more valid results.

**Interpreting sex, gender, and their effects as biopsychosocial**

ORIGINAL UNEDITED MANUSCRIPT

Even in a hypothetical future with better measurement and data sets containing both biological and psychosocial measures, health would involve interacting sexed and gendered processes. This "entanglement" of sex and gender should be assumed, absent strong evidence to the contrary (16). Social gender can shape biology, just as biological sex can have psychosocial effects, and these possibilities can be difficult to disaggregate (1,16). Personal behavior, social interaction, and environment can alter sex hormones (104), even in ways that counter gendered assumptions about biology. For example, infant cries increased testosterone in men, with subsequent decrease dependent on their success at calming the infant (105). The initiation and continuation of the sexed condition of pregnancy is predicated on gendered behaviors/roles and policies, and in turn influences highly gendered pregnancy-related social interactions. Thus, while sex and gender approaches to epidemiology inform causal understanding, they would rarely be expected to produce estimates of pure effects cleanly distinct from other domains and dimensions. This does not make such distinctions less useful, but requires appropriate interpretation. For example, research has shown interaction between gender roles and sex in allostatic load (106), demonstrating multidimensional effects and informing causal understanding; yet more specific interacting causal pathways remain possible. This necessitates interpretation within a biopsychosocial approach (107), one that accepts that biology and physiology both shape and are shaped by social context and behavior.

ORIGINAL UNEDITED MANUSCRIPT

**CONCLUSION**

Together, the considerations presented provide an opportunity for epidemiologists to strengthen research design, measures, and interpretation (Table 3). A fuller integration of sex and gender in epidemiology serves to better identify health disparities and causal processes that generate them. Including analysis of relevant sex- or gender-associated factors can help prevent problematic overdetermination of sex or gender and “pink vs. blue” understandings of health, and better identify potential intervention targets. Finally, these distinctions in epidemiological research serve to produce more specific observations which refine theory to further inform research both within epidemiology and across disciplines.

**Table 1. Sex and gender multidimensionality at the individual level: A conceptual tool for epidemiologists**

| Dimension | Description | Potential change over life course |
| --- | --- | --- |
| | *Sex* | |
| Chromosomal sex | Karyotype (XX, XY, XO, XXY); chimerism | No [a] |
| Sex assigned at birth | Recorded on initial birth record; generally genital phenotype | No |
| Hormonal milieu | Endogenous and exogenous sex steroids | Yes |
| Reproductive sex | Gametes | Yes |
| Organ-specific status | Presence of a sex-specific organ (e.g., uterine status) | Yes |
| Sexed physiology | Sexed physiological measures (e.g., lactation, semen production) | Yes |
| Intersex status | Reported presence of intersex conditions generally, or a specific condition | Yes |
| Pregnancy | Temporary pregnancy-specific anatomy (e.g., placenta) and physiology (e.g., transplacental microtransfusion) | Yes |
| | *Gender* | |
| Gender identity | Personally held sense of one's gender as man/boy, woman/girl, another cultural gender, trans, non-binary, etc. | Yes |
| Intersex identity | Personally held identification as intersex | Yes |
| Lived gender | Expressed gender, or how one presents oneself in day-to-day life | Yes |
| Gender role | Gendered social, ceremonial, or work roles, including men's, women's and other culturally specific roles | Yes |
| Metaperceived gender | Gender one knows others perceive or treat them as, including perception as gender minority | Yes |
| Masculinity and/or femininity | Social and historically situated norms regarding men/boys and girls/women | Yes |
| Internalized gender stigma | Internalized beliefs regarding one's own sex/gender (e.g, internalized cisnormativity,[b] internalized misogyny [c]) | Yes |
| Enacted gender stigma | Personal experiences of sexism, transphobia, or | Yes |

ORIGINAL UNEDITED MANUSCRIPT

| | | |
|---|---|---|
| / discrimination | homophobia | |
| Gender ideology | Attitudes toward, or agreement with, a cultures gender norms | Yes |
| | *Sex/gender* | |
| Administrative sex/gender | Undifferentiated sex/gender indicator within administrative data | Yes |
| Undifferentiated survey item sex/gender | Survey item recorded by participant based on unclear distinction | Yes |
| Computer (AI)-classified sex/gender | Algorithmically assigned gender categories or probabilities | Yes |
| Researcher-perceived sex/gender | Survey item recorded by researcher based on appearance, name or voice | Yes |
| | *Gender minority cross-classifications* [d] | |
| Gender identity ≠ birth-labelled sex | Umbrella classification for all whose gender identity differs from sex assigned at birth | Yes |
| Lived gender ≠ birth-labelled sex | Umbrella classification for all whose lived gender differs from sex assigned at birth | Yes |
| | *Sex- or gender-associated factors* | |
| Biological, psychological, behavioral, interpersonal, and social factors [e] | Factors associated with sex/gender that are not themselves dimensions of sex or gender (e.g., gene expression, body weight, risk taking, age at sexual debut, structural sexism) | Yes |

AI, artificial intelligence.

a. Two exceptions here are loss of Y chromosomes and some forms of microchimerism. Mosaic loss of Y chromosomes is common and increases with age. While twin-to-twin and maternal-fetal transfer in utero may result in sustained microchimerism, so too may microchimerisms produced later in the life course through fetal-maternal transfer, organ or bone marrow transplantation, or blood transfusion.
b. Internalization of idea that all dimensions of sex and gender should be concordant within oneself.
c. Internalized negativity toward one's own femaleness, women's roles, or femininity.
d. These represent broad cross-classifications; gender minority identities, roles, expression, metaperception, and internalization are included under Gender.
e. While sex/gender-associated factors are not dimensions of sex or gender *per se*, they may explain observed sex or gender differences. As biological, psychological/behavioral, and interpersonal or social causation may interact, the distinction between sex and gender in these associations is often not always clear; for example, body weight is a function of both sexed biology such as height and of social behaviors such as dieting and exercise.

ORIGINAL UNEDITED MANUSCRIPT

**Table 2. Case study 2: Hypothesized sexed and gendered factors in COVID-19 morbidity and mortality**

| Dimensions and Factor | Infection[a] | Diagnosis | Prognosis[b] |
|---|---|---|---|
| *Sex* | | | |
| Chromosomal sex | | | |
| Sex chromosome-linked genes (e.g., ACE2 receptor) | X[67,75,108] | | X[74,77,108] |
| Hormonal milieu | | | |
| Androgens; androgen-deprivation therapy | X[58,71] | | X[109] |
| Estrogens | | | X[58,70,71] |
| Pregnancy | | | |
| Altered immune response during pregnancy | X[110] | | |
| *Gender* | | | |
| Gender role | | | |
| Caregiver to ill family members | X[67] | | |
| Frontline health care worker role | X[67,77] | | |
| *Gender minority cross-classifications* | | | |
| Lived gender ≠ birth-labelled sex | | | |
| Avoidance of health care settings | | X[77] | |
| *Sex/gender-associated factors* | | | |
| Biological factors | | | |
| Innate and adaptive immune response | X[67,75,77,108] | | X[74,75,111] |
| Cardiovascular disease | | | X[77,108] |
| Behavioral factors | | | |
| Health care seeking | | X[67,77,111] | X[67,75] |
| Tobacco smoking | | | X[75,77,112] |
| Preventive behaviors (hand-washing, mask wearing) | X[67,77] | | |
| Social factors | | | |
| Structural discrimination in design/fit of PPE[c] | X[77] | | |

ACE2, angiotensin converting enzyme 2; PPE, personal protective equipment.

a. Infection includes factors that may increase the probability of exposure or the probability of infection once exposed.
b. Prognosis includes severe disease (often indicated by hospitalization or intensive care) or death.
c. Personal protective equipment (PPE) worn by health care workers and other essential workers is most often designed based on measurements from young male military recruits in the U.S. in the mid-20th century.

ORIGINAL UNEDITED MANUSCRIPT

**Table 3. Recommendations for researchers**

| Role | Guidance |
|---|---|
| Study design | Specify dimension(s) of biological sex and/or social gender of theoretical relevance to the study. |
| | If constructing a causal model (e.g., a directed acyclic graph), diagram each dimension as a separate node to allow dimensions to affect others. |
| | Consider direct or mediated causal pathways for sex- and gender-associated factors that may be driving sex or gender differences. |
| | Draw on theoretical frameworks that provide a multi-level, intersectional and/or biopsychosocial perspective. |
| Data collection | Review recent research on measurement of relevant dimensions of sex and/or gender. |
| | In multi-user population data sets, consider collecting multiple dimensions of sex and/or gender. |
| Data analysis | Where possible, differentiate between effects of multiple sex or gender measures, as well as sex- and gender-associated factors. |
| | Consider conducting research on the validity of proxy measures for dimensions of sex and gender. |
| Knowledge translation | If an existing measure (e.g., a nondifferentiated sex/gender measure from administrative data) does not match the relevant dimension, explicitly acknowledge that it is a proxy for the specified measure of interest. |
| | Report available information on how an existing sex, gender, or nondifferentiated sex/gender measure performs as a proxy, including whether its performance may vary across population subgroups such as race, ethnicity or social class. |
| | Where applicable, acknowledge limitations of sex and gender measurement. |
| | Interpret sex and gender results in a biopsychosocial context, acknowledging the ways sexed biology shapes and is shaped by gendered social and behavioral factors. |

ORIGINAL UNEDITED MANUSCRIPT

**Figure 1A. Case study 1: Estimates for sensitivity of database-encoded sex/gender as a proxy for uterine status**

**Figure 1B. Case study 1: Estimates for specificity of database-encoded sex/gender as a proxy for uterine status**

Legend: B20 = Black non-Hispanic, age 20-39; B40 = Black non-Hispanic, age 40-59; B60 = Black non-Hispanic, age ≥ 60; W20 = White non-Hispanic, age 20-39; W40 = White non-Hispanic, age 40-59; W60 = White non-Hispanic, age ≥ 60. Estimates were produced by combining data from the National Health and Nutrition Examination Survey (2013-2018) with published estimates from the 2010 U.S. Census, 2015 U.S. Trans Survey, and a Williams Institute report (see Web Appendix 2 for details).

ORIGINAL UNEDITED MANUSCRIPT

A)

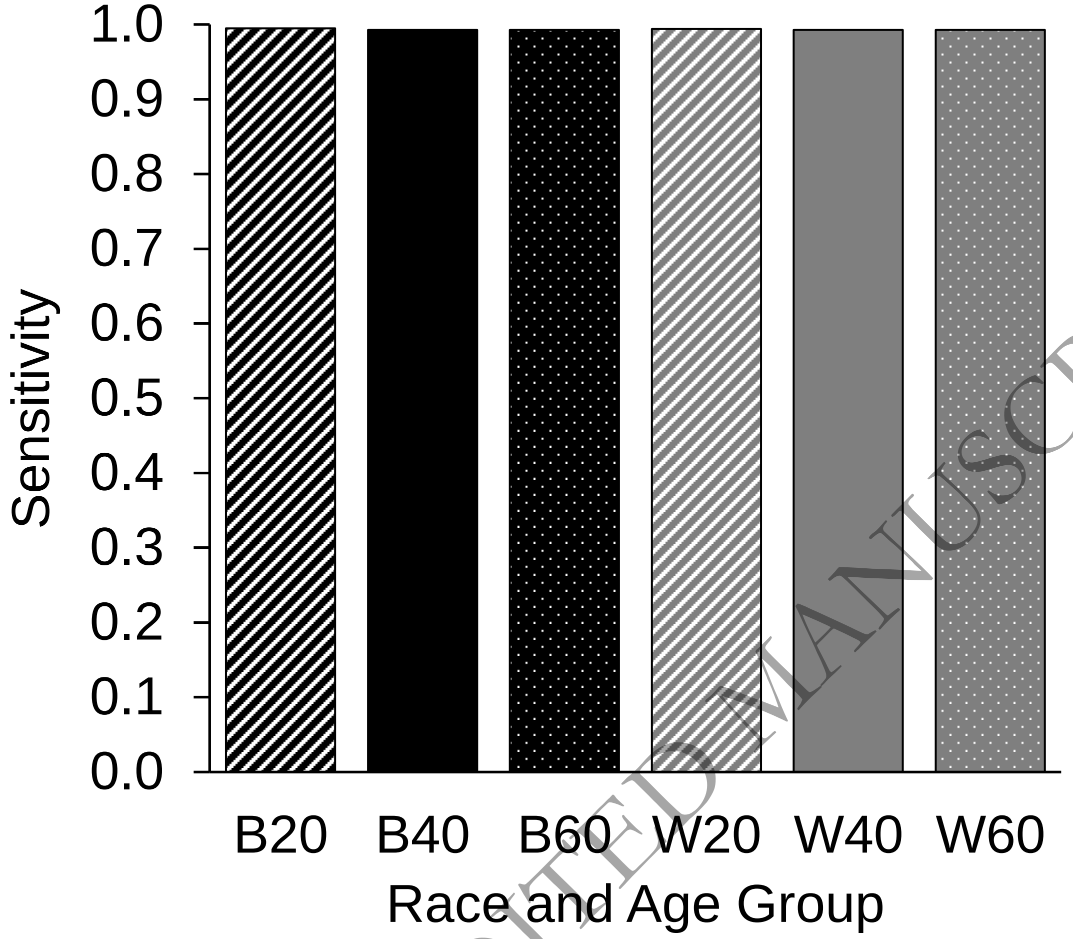

B)

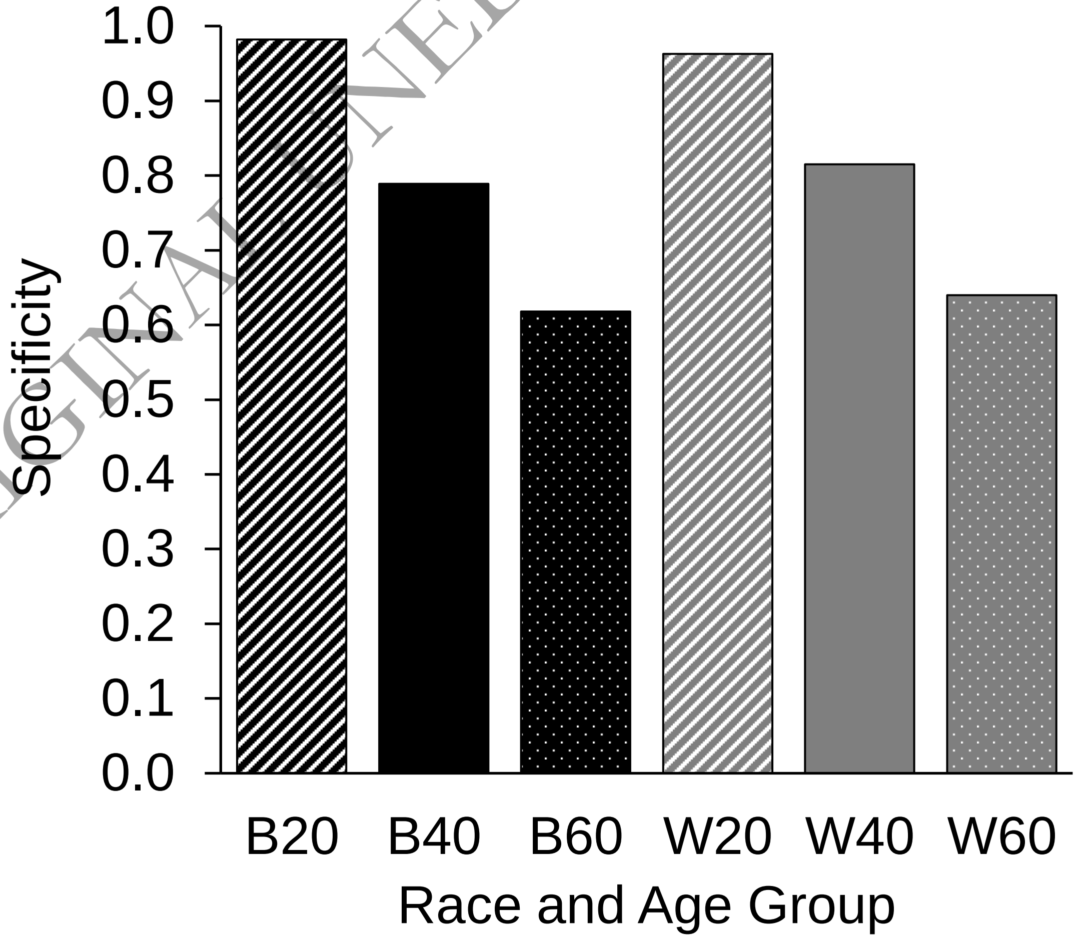

ORIGINAL UNEDITED MANUSCRIPT

## Supplementary Material

Web_Material_kwac173

