# Supplementary material for: Sex and Gender Multidimensionality in Epidemiologic Research
Source: Am J Epidemiol. 2022 Oct 4;192(1):122–32. doi: 10.1093/aje/kwac173 (PMC9619685; doi:10.1093/aje/kwac173)
Supplement: Web_Material_kwac173 [file web_material_kwac173.pdf]

Web Material

Sex and Gender Multidimensionality in Epidemiologic Research

Greta R. Bauer

Contents

Web Appendix 1. Original NHANES analysis of hysterectomy prevalences 2

Web Table 1. Proportion with hysterectomy, Black and white non-Hispanic women aged 20 and older, NHANES 2013-2018 2

Web Appendix 2. Calculations for validity estimates in Case Study 1 2

Web Table 2. 2x2 table for estimating sensitivity and specificity of database-encoded sex/gender as a proxy for uterine status in each stratum within illustrative example 2

Web Table 3. Resulting estimates for the six race by age strata in the illustrative example 3

Web Table 4. Data components used to produce sensitivity and specificity estimates 3

References 4

Web Appendix 1. Original NHANES analysis of hysterectomy prevalences

Three data releases from the National Health and Nutrition Examination Study (NHANES)[1] were merged to combine data collected from 2013 through 2018 (n=29,400).

Data analysis was conducted using the SURVEYFREQ procedure in SAS version 9.4.1.[5] All analyses were weighted. While not used in the sensitivity and specificity calculations, the 95% confidence intervals in the table below were estimated using Tayler series linearization. A domain analysis was conducted using data from n=4,477 Black or white non-Hispanic women aged 20 and older for whom data on hysterectomy status were available.

Web Table 1. Proportion with hysterectomy, Black and white non-Hispanic women aged 20 and older, NHANES 2013-2018

		Hysterectomy	
**Race*age group**	**n**	**Weighted %**	**95% CI**
Black non-Hispanic women, 20-39 years	523	1.2449	(0.1640, 2.3257)
Black non-Hispanic women, 40-59 years	584	25.3206	(20.5126, 30.1285)
Black non-Hispanic women, ≥ 60 years	527	49.1320	(44.5752, 53.6888)
White non-Hispanic women, 20-39 years	827	3.2815	(1.7486, 4.8145)
White non-Hispanic women, 40-59 years	865	21.3077	(17.3066, 25.3087)
White non-Hispanic women, ≥ 60 years	1151	44.6820	(40.1899, 49.1742)

Web Appendix 2. Calculations for validity estimates in Case Study 1

Web Table 2. 2x2 table for estimating sensitivity and specificity of database-encoded sex/gender as a proxy for uterine status in each stratum within illustrative example

		True Value		
		**Uterus +**	**No Uterus -**	
**Measure (test result)**	**Database-encoded Woman +**	P_W [$(1 - P_T)(1 - P_{H\|CW})$]	P_W [$(1 - P_T)(P_{H\|CW}) + P_T$]	P_W
	**Database-encoded Man -**	P_M [$(P_T)(1 - P_{H\|TM})$]	P_M [$(1 - P_T) + (P_T)(P_{H\|TM})$]	P_M
		P_W [$(1 - P_T)(1 - P_{H\|CW})$] + P_M [$(P_T)(1 - P_{H\|TM})$]	P_W [$(1 - P_T)(P_{H\|CW}) + P_T$] + P_M [$(1 - P_T) + (P_T)(P_{H\|TM})$]	$P_W + P_M$

Here the data coding takes two categories for women and men, with $P_W + P_M = 1.0000$.

Stratum-specific estimates

P_W = proportion database-encoded women

P_M = proportion database-encoded men

$P_{H|CW}$ = proportion of cisgender women who have had hysterectomies

$P_{H|TM}$ = proportion of trans men who have had hysterectomies

P_T = proportion transgender (assumed same, but can adjust if different among women and men)

A = P_W [$(1 - P_T)(1 - P_{H|CW})$]

B = P_W [$(1 - P_T)(P_{H|CW}) + P_T$]

C = P_M [$(P_T)(1 - P_{H|TM})$]

D = P_M [$(1 - P_T) + (P_T)(P_{H|TM})$]

A = cisgender women without hysterectomy (no trans women with uteri)
B = cisgender women who have had hysterectomy + trans women
C = trans men with uteri
D = cisgender men and trans men without uteri

Web Table 3. Resulting estimates for the six race by age strata in the illustrative example

Stratum	P_W	P_M	P_T	P_{HCW}	P_{HTM}	A	B	C	D	Sensitivity	Specificity
Black non-Hispanic 20-39	0.4967	0.5033	0.0058	0.012449	0.08	0.487672	0.009028	0.002686	0.500614	0.994523	0.982285
Black non-Hispanic 40-59	0.5084	0.4916	0.0058	0.253206	0.08	0.377468	0.130932	0.002623	0.488977	0.993099	0.788788
Black non-Hispanic 60+	0.5543	0.4457	0.0050	0.491320	0.08	0.280552	0.273748	0.002050	0.443650	0.992745	0.618415
White non-Hispanic 20-39	0.4967	0.5033	0.0058	0.032815	0.08	0.477614	0.019086	0.002686	0.500614	0.994408	0.963276
White non-Hispanic 40-59	0.5084	0.4916	0.0058	0.213077	0.08	0.397751	0.110649	0.002623	0.488977	0.993448	0.815470
White non-Hispanic 60+	0.5543	0.4457	0.0050	0.446820	0.08	0.305095	0.249205	0.002050	0.443650	0.993325	0.640321

Web Table 4. Data components used to produce sensitivity and specificity estimates

Description	Estimate	Source
Hysterectomy prevalence, Non-Hispanic Black women aged 20-39 [a]	0.012449	NHANES[1] 2013-2018, original data analysis
Hysterectomy prevalence, Non-Hispanic Black women aged 40-59 [a]	0.253206	NHANES[1] 2013-2018, original data analysis
Hysterectomy prevalence, Non-Hispanic Black women aged 60+ [a]	0.491320	NHANES[1] 2013-2018, original data analysis
Hysterectomy prevalence, Non-Hispanic white women aged 20-39 [a]	0.032815	NHANES[1] 2013-2018, original data analysis
Hysterectomy prevalence, Non-Hispanic white women aged 40-59 [a]	0.213077	NHANES[1] 2013-2018, original data analysis
Hysterectomy prevalence, Non-Hispanic white women aged 60+ [a]	0.446820	NHANES[1] 2013-2018, original data analysis
Proportion of U.S. population that is transgender (age 25-64)	0.0058	Williams Institute, University of California Los Angeles [2] (used for ages 20-59 in estimates)
Proportion of U.S. population that is transgender (age 65+)	0.0050	Williams Institute, University of California Los Angeles [2] (used for ages 60+ in estimates)
Proportion of transmasculine persons in U.S. who have had hysterectomies [b]	0.08	U.S. Trans Survey [3]
Proportion of U.S. population that are women, age 20-39 [c]	0.4967	2010 U.S. Census [4]
Proportion of U.S. population that are women, age 40-59 [c]	0.5084	2010 U.S. Census [4]
Proportion of U.S. population that are women, age 60+ [c]	0.5543	2010 U.S. Census [4]

a. Given the very small proportion of women who are trans or without uteri due to intersex conditions (not identified in data set), this serves as an estimate for cisgender women born with uteri.
b. Age and ethnoracial breakdowns not included in published documents.
c. Ethnoracial breakdowns not included in published documents.

References

1. National Center for Health Statistics. NHANES - National Health and Nutrition Examination Survey. Published February 23, 2021. Accessed March 4, 2021. https://www.cdc.gov/nchs/nhanes/index.htm

2. Flores AR, Herman JL, Gates GJ, Brown TNT. How Man Adults Identify as Transgender in the United States? *2016*. Published online 2016:13.

3. James SE, Herman JL, Rankin S, Keisling M, Mottet L, Anafi M. *The Report of the 2015 U.S. Transgender Survey*. The National Center for Transgender Equality; 2016:302. Accessed June 12, 2020. https://www.transequality.org/sites/default/files/docs/USTS-Full-Report-FINAL.PDF

4. Howden LM, Meyer JA. *Age and Sex Composition: 2010*. U.S. Census Bureau; 2011:16. Accessed March 3, 2021. https://www.census.gov/prod/cen2010/briefs/c2010br-03.pdf

5. *SAS*. SAS Institute
